# Race-specific associations between health-related quality of life and cellular aging among adults in the United States: evidence from the National Health and Nutrition Examination Survey

**DOI:** 10.1007/s11136-017-1610-9

**Published:** 2017-06-08

**Authors:** Rumana J. Khan, Samson Y. Gebreab, Pia R. Crespo, Ruihua Xu, Amadou Gaye, Sharon K. Davis

**Affiliations:** Genomics of Metabolic, Cardiovascular and Inflammatory Disease Branch, Social Epidemiology Research Unit, National Human Genome Research Institute, National Institutes of Health, 10 Center Drive, Room 7N316, MSC 1644, Bethesda, MD 20892 USA

**Keywords:** Health-related quality of life, Perceived health status, Leukocyte telomere length, Aging, Race

## Abstract

**Purpose:**

Poor health-related quality of life (HRQOL) could lead to higher morbidity and mortality through telomere attrition or accelerated cellular aging. We conducted a cross-sectional analysis to examine the relationship between four dimensions of HRQOL and leukocyte telomere length (LTL) among a nationally representative sample of 3547 US adults (≥20 years) using the data from the 2001–2002 National Health and Nutrition Examination Survey.

**Method:**

We used HRQOL survey information collected on individuals’ self-rated general health, recent physical health, recent mental health, and recent activity limitation. Telomere length was assessed using quantitative polymerase chain reaction. Multiple linear regressions were used to estimate the relationship between each dimension of HRQOL and log-transformed values of LTL with adjustment for sample weights and design effects.

**Results:**

HRQOL-race interactions were significant, and the results were stratified by race. After controlling for demographic factors, disease conditions, and lifestyle variables, worse general health was significantly associated with shorter LTL for Blacks (coefficient, β: −0.022, 95% Confidence Interval, 95% CI: −0.03 to −0.01), but not for Whites or Mexican Americans. Unwell physical health was associated with shorter telomere length for Whites (β: −0.005, 95% CI: −0.01 to −0.001) only. Unwell mental health showed no significant association with LTL in any race.

**Conclusions:**

Although longitudinal studies are needed to prove causality, our findings suggest that HRQOL could be associated with LTL shortening. We also found a possible racial difference in this association and recommend additional multiethnic studies to confirm this and to understand the reasons and consequences of this difference.

**Electronic supplementary material:**

The online version of this article (doi:10.1007/s11136-017-1610-9) contains supplementary material, which is available to authorized users.

## Introduction

Health-related quality of life (HRQOL), which is defined as “perceived or self-rated physical and mental health over time,” provides a subjective weighting of health conditions and provides an indication of the economic and psychosocial burden of an individual’s level of health that are often overlooked by traditional disease measures [1, 2]. While measures of disease and mortality are crucial, they indicate little about other important aspects of an individual’s level of health, including related economic and psychosocial elements such as the impact of health problems on quality of life, pain and suffering, social discrimination, social and role functioning, economic burden, health perceptions, and life satisfaction. [2]. To bridge this gap between traditional health measures and overall wellbeing, the concept of HRQOL was put forward by the World Health Organization as comprehensive, easy, and low-burden measures to explore health-related quality of life as broader measures of population health status [2, 3].

Studies show that inferior HRQOL predicts morbidity and mortality even after accounting for important health risk factors [4–11]. However, the molecular or cellular mechanisms through which HRQOL may contribute to poorer health outcomes are not well understood. Several studies have indicated shorter leukocyte telomere length (LTL) to be associated with increased risk of mortality and various disease conditions, including immune response and infection-related diseases, diabetes, hypertension, cancer, and atherosclerosis and other cardiometabolic disorders, dementia, and cancers [12–18]. Therefore, LTL may provide the biological link between economic and psychosocial burden related to HRQOL and different health outcomes.

Human telomeres are nucleoprotein structures located at the ends of chromosomes and protect them from degradation, fusion, and recombination in somatic cells [19, 20]. LTL generally shortens progressively with every cell division over the lifespan and, thus, telomere shortening is strongly associated with age in most somatic tissues and telomere length typically declines with age [21, 22]. Although genetic and epigenetic factors play important roles to determine early life LTL, evidence suggests that multiple environmental factors, which lead to cellular stress, oxidation, and inflammation, may also affect LTL in adulthood [23, 24]. For example, prolonged states of mental stress and adverse psychological conditions, such as depression and post-traumatic stress disorders, have been found to be associated with shorter telomere length in many occasions [25–30]. No studies of which we are aware have investigated the exact association between HRQOL with LTL. But comparable indicators such as various forms of chronic psychosocial stressors—life stress, low socioeconomic status, racial discrimination, social interactions, perceived social control across different health-relevant domains, and perceived unfair treatment—were shown to be associated with telomere shortening in several studies [31–40]. The underlying mechanisms by which these factors affect LTL are not fully understood, but as stated earlier, they may contribute to shorter LTL by increasing oxidative stress and inflammation—two biological mechanisms that are known to cause accelerated LTL shortening [23, 24].

It is often suggested that low HRQOL is indicative of low psychosocial functioning and aggravated psychosocial stress [41]. Wolkowitz et al. observed that biological derangements seen in chronic stress (e.g., inflammation, oxidative stress, and perhaps changes in steroids) are associated with, and may cause, telomere shortening through greater cellular and genomic damage, and depleted repair and protection process [42, 43]. Therefore, it is possible that HRQOL could lead to multiple biological risk factors, higher disease morbidity, and mortality, partly through accelerating premature aging of cells. Since HRQOL may impact health by altering health behaviors, it is also possible that rather than being causally related, HRQOL leads to telomere shortening through third factors common to both, such as life style factors like lack of physical activity, cigarette smoking, alcohol drinking, poor sleep, and poor nutrition. [44–46]. Therefore, understanding the relationships between HRQOL and LTL has the potential to elucidate if the impact of quality of life-related factors on life-shortening diseases could partly be explained by telomere biology. Additionally, this can also provide insight into if interventions targeting the improvement of quality of life-related factors might simultaneously be effective in aiding telomere maintenance or lengthening. Because HRQOL has the ability to capture important economic and psychosocial elements beyond mere health status, this will also enable us to have an idea about the impact of these elements on telomere shortening. Moreover, it is also important to know whether there are differences in this association by race, because, although the majority of studies reported a pattern of Blacks having longer LTL than Whites [47–49], cumulative exposure to multiple sources of psychosocial stressors over the lifecourse has been suggested as possible contributors to faster LTL shortening with age in Blacks than in Whites [50].

In this study, we hypothesized that poorer perceptions of quality of life were associated with greater cellular aging or shorter LTL, and examined the cross-sectional associations between four dimensions of HRQOL and LTL by race among 3547 nationally representative sample of US adults (≥20 years) using the data from the 2001–2002 National Health and Nutrition Examination Survey (NHANES) after adjusting for individual-level risk factors.

## Methods

### Data source

We used the data of the 2001–2002 NHANES, which was conducted between January 2001 and December 2002 in 5411 adult individuals (age ≥20 years). NHANES is a nationally representative survey that uses a complex, stratified, multistage probability sampling design. The detailed description of the survey methodologies and analytic guidelines have been reported elsewhere [51]. In total, our analysis included 3547 White, Black, and Mexican American participants (flowchart: supplementary chart 1). The NHANES datasets are de-identified and available in the public domain. This study was exempted from human subject review by the National Institutes of Health Office for Human Subjects Research Protections (OHSRP13100). The data analysis was done in 2016.

### Variables

HRQOL was determined using the validated four-dimension HRQOL-4 questionnaire, developed by the Centers for Disease Control and Prevention [52]. This HRQOL-4 questionnaire has demonstrated reliability and validity for population health surveillance [52–55]. The survey asked participants about their overall perceived general health, and the number of unwell days in the past 30 days due to poor physical health, mental health, or activity limitation. Perceived or self-rated health was ascertained by response to the question “In general, would you say your health is: excellent, very good, good, fair, or poor?” Given the low frequency of the “excellent” and “poor” responses, we combined “excellent” with “very good” and “fair” with “poor.” Thus, the scales were re-categorized into three groups: “excellent” (excellent + very good), “good,” and “poor” (fair + poor), with “excellent” being the reference group for analysis. Physical unwell days were defined by the question “Now thinking about your physical health, which includes physical illness and injuries, for how many days during the past 30 days was your physical health not good?”; mental unwell days by “Now thinking about your mental health, which includes stress, depression, and problems with emotions, for how many days during the past 30 days was your mental health not good?”; and limited activity days by “During the past 30 days, for about how many days did poor physical or mental health keep you from doing your usual activities, such as self-care, work, or recreation?” The total number of unwell days (physical and mental and limited activity) was grouped into 0, 1–15, and 16–30 days, where 0 day served as the reference group [56]. The detailed survey questions and their re-categorization are presented in Supplementary chart 2. The Cronbach’s alpha was 0.713 for the HRQOL-4 instrument [57].

All participants aged 20 years and older, who were examined in 2001–2002 and who had blood collected for DNA purification, were eligible for LTL quantification. LTL relative to standard reference DNA (T/S ratio) was measured using the quantitative polymerase chain reaction method [58]. The formula to convert T/S ratio to base pairs (bp) was 3274 + 2413 × (T/S) [58, 59]. Detailed analytical methods are documented on the NHANES website [51, 60]. The quality control procedure is summarized in the Supplementary section.

### Other covariates

Self-reported race information was used. Participants were asked what race (“White,” “Black,” “Mexican American,” or “Other race,” which included mixed race, Asian, and other Hispanic) they belonged to. For the current analysis, the “Other race” category was not considered, because respondents only formed about 6% of the total available sample and, more importantly, their race was not as clearly specified as other major races (flowchart: supplementary chart 1). Age was calculated in years. Educational attainment was dichotomized as less than high school and high school or more. Marital status was categorized as married/partnered and not married/partnered. Smokers were defined as smoking ≥100 cigarettes during lifetime. Participants were considered physically active if they were involved in vigorous activity over the past 30 days (yes/no). Alcohol consumption was defined as having at least 12 drinks in any one year. Body mass index or BMI (weight in kg/height in meter^2^) was calculated using height and weight measurements. The cut point of 30 or higher was defined as obesity [61]. Information on existing chronic disease conditions (yes/no) including diabetes, high blood pressure, congestive heart failure, and cancer or malignancy was obtained.

### Statistical analysis

To account for the complex survey design (strata and primary sampling unit indicators), we used Stata Version 12 software’s “svy” survey data commands (Stata Corp., College Station, TX) and applied NHANES sample weights for the genetic subsample for all analysis [51, 60]. These weights additionally account for survey non-response (note: supplementary chart 1) [51, 60]. Each variable was assessed for outliers and normal distributions. Weighted means with 95% confidence interval (95% CI) and weighted proportions with 95% CI were calculated for continuous and categorical variables, respectively, within each racial category. Additionally, age-adjusted LTL was derived using regression coefficient and summarized by race. For descriptive results, non-overlapping 95% CIs indicate statistical significance.

Multiple linear regressions were used to estimate the relationship between LTL and each of the four dimensions of HRQOL (perceived general health, unwell days due to poor physical health, poor mental health, or activity limitation). We sequentially controlled for demographic factors (age, sex, education, marital status), disease conditions (cancer, hypertension, diabetes, obesity, heart failure), and lifestyle variables (smoking, physical activity, and alcohol intake), respectively, in models 1, 2, and 3. Given prior evidence of race/ethnic differences in perceived health status and LTL, we estimated models stratified by race [50]. We also tested for the statistical interactions between four dimensions of HRQOL and race on LTL. To examine the variation by gender, interaction between HRQOL and gender was also checked. To evaluate the interactions, we added main effects and cross-product terms to the regression after adjusting for all the variables mentioned previously. *P* value for each interaction term and *F* tests comparing full and reduced models (with and without the interaction term) were used to test the statistical significance of the interaction terms. LTL was transformed by natural logarithm before regression to improve normality and stabilize the variance. Therefore, we report the percentage change (unstandardized regression coefficient) in the average value of LTL (the outcome variable) for each dimension of HRQOL. Because of the exploratory nature of all the analyses, no correction for multiple testing was conducted [62, 63] and statistical significance was defined as a *P* value < 0.05.

## Results

We found no evidence of effect modification by sex affecting associations between HRQOL and LTL. Consistent with our hypothesis, HRQOL-race interaction remained significant (findings summarized in the supplementary section) for recent days of unwell physical health (*p* < 0.05) and perception of self-rated general health (*p* value < 0.05); hence, we present our result stratified by race. The weighted distributions of study population characteristics from the NHANES of 2001–2002 are shown in Table 1. Of the 3547 participants with ages ranging from 20 to 85 (mean age 49) years, 2090 (58.92%) were White, 669 (18.86%) Black, and 788 (22.22%) Mexican Americans. Whites were older (mean age 46.89, 95% CI: 45.71–48.08) than Blacks (mean age 41.99, 95% CI: 40.42–43.56) or Mexican American (37.57, 95% CI: 35.99–39.14). They were also more educated than the other two races. About 70% of the Whites and Mexican Americans were either partnered or married compared to only 47% of the Blacks. Whites were more likely to smoke, drink alcohol, and be involved in physical activities than the participants of the other two races. Blacks had higher prevalence of obesity, diabetes, hypertension, and congestive cardiac failure than Whites or Mexican Americans, but were less likely to have had cancer than Whites. Blacks had longer LTL (mean 6001.31 bp, 95% CI: 5858.69–6143.92) than Whites (mean 5854.34 bp, 95% CI: 5741.23–5967.45) or Mexican Americans (mean 5900.65 bp, 95% CI: 5818.87–5982.42). However, according to the age-adjusted predicted values of LTL, Mexican Americans had longer LTL than Whites or Blacks.

**Table 1:** Demographic characteristics and telomere length of US adult population by race (National Health Interview Survey, 2001–2002, *N* = 3547)

	White (*N* = 2090)	Black (*N* = 669)	Mexican American (*N* = 788)
Age in years^a^	46.89 (45.71–48.08)	41.99 (40.42–43.56)	37.57 (35.99–39.14)
Gender			
Male	49.96 (48.51–51.48)	44.98 (41.21–48.74)	51.78 (48.67–54.89)
Female	50.03 (48.58–51.48)	55.01 (51.25–58.78)	48.21 (45.10–51.32)
Education			
Less than high school	12.92 (9.94–15.90)	32.38 (25.26–39.49)	51.47 (45.36–57.59)
More than high school	87.07 (84.09–90.05)	67.61 (60.50–74.73)	48.52 (42.40–54.63)
Marital status			
Married or partnered	69.57 (66.99–72.16)	47.64 (43.98–51.23)	70.49 (65.53–75.27)
Telomere length in base pairs^a,b^	5854.34 (5741.23–5967.45)	6001.31 (5858.69–6143.92)	5900.65 (5818.87–5982.42)
Age-adjusted telomere Length in base pairs^a,b,c^	5864.35 (5845.96–5882.75)	5940.27 (5916.00–5964.55)	6008.86 (5984.47–6033.24)
Smoked at least 100 cigarettes in life	51.68 (46.35–57.01)	43.77 (38.82–48.72)	42.00 (36.92–47.08)
Had at least 12 alcohol drinks in any one year	76.59 (67.26–85.92)	59.65 (52.48–66.83)	67.16 (62.45–71.88)
Vigorous physical activity over past 30 days	39.38 (35.26–43.49)	30.94 (26.25–35.63)	33.75 (29.29–38.21)
Obesity^d^	29.57 (26.28–32.85)	40.56 (36.16–44.95)	29.91 (24.59–35.24)
Known diabetes	6.82 (5.74–7.90)	10.75 (8.04–13.45)	8.72 (7.62–9.81)
Known hypertension	25.71 (22.82–28.59)	36.88 (33.62–40.14)	11.96 (10.38–13.55)
Known congestive heart failure	1.98 (1.05–2.92)	2.91 (1.41–4.41)	1.09 (0.3–1.88)
Known cancer or malignancy	9.97 (8.09–11.86)	3.24 (2.37–4.11)	1.79 (0.73–2.86)

Analyses were done with adjustment for sample weights and design effects

Data represent percentage (95% confidence interval), except where noted

^a^Mean (95% confidence interval)

^b^Calculated using formula “3274 + 2413 × T/S ratio (telomere length relative to standard reference DNA)”

^c^Derived using regression coefficient for age

^d^Defined as body mass index (weight in kg divided by height in meter square) of 30 or higher

Table 2 presents race-specific proportions of participants reporting each dimension of HRQOL. Whites were more likely to report better general health than the other two races. Approximately 58% of whites reported having either excellent or very good general health compared to 37.67% of Blacks and 28.39% of Mexican Americans. This pattern, however, was not observed for measures related to “unwell days.” Individuals of all three races reported similar proportions of “unwell days” (0, 1–15, and 16–30 days) due to physical health, mental health, and activity limitation.

**Table 2:** Proportions of responses to four dimensions of health-related quality of life (HRQOL) questions of US adult population by race (National Health Interview Survey, 2001–2002, *N* = 3547)

	White (*N* = 2090)	Black (*N* = 669)	Mexican American (*N* = 788)
General health			
Excellent/very good	57.53 (52.74–62.31)	37.67 (29.54–45.80)	28.39 (24.17–32.60)
Good	31.01 (26.87–35.15)	37.48 (32.00–42.96)	41.21 (36.66–45.77)
Poor/fair	11.45 (9.44–13.46)	24.84 (17.79–31.88)	30.39 (26.44–34.33)
Unwell physical health during the past 30 days			
0 days	62.43 (58.97–65.89)	60.65 (54.41–66.89)	65.25 (60.33–70.16)
1–15 days	31.01 (27.69–34.32)	30.61 (26.01–35.02)	29.20 (24.11–34.29)
16–30 days	6.54 (5.54–7.55)	8.73 (4.54–12.91)	5.54 (3.73–7.34)
Unwell mental health during the past 30 days			
0 days	60.37 (56.38–64.36)	59.08 (52.64–65.52)	60.30 (57.44–63.16)
1–15 Days	32.44 (29.15–35.72)	30.35 (24.65–36.05)	34.36 (31.38–37.33)
16–30 days	7.18 (5.56–8.79)	10.56 (7.22–13.89)	5.33 (4.12–6.54)
Activity limitation during the past 30 days			
0 days	81 (80.21–83.74)	79.47 (73.92–85.02)	83.52 (80.64–86.41)
1–15 Days	15.59 (13.77–17.40)	15.74 (11.35–20.14)	14.18 (11.56–16.79)
16–30 days	2.43 (1.51–3.35)	4.44 (1.66–7.88)	2.29 (0.98–3.59)

Analyses were done with adjustment for sample weights and design effects

Data represent percentage (95% confidence interval)

**Fig. 1:**
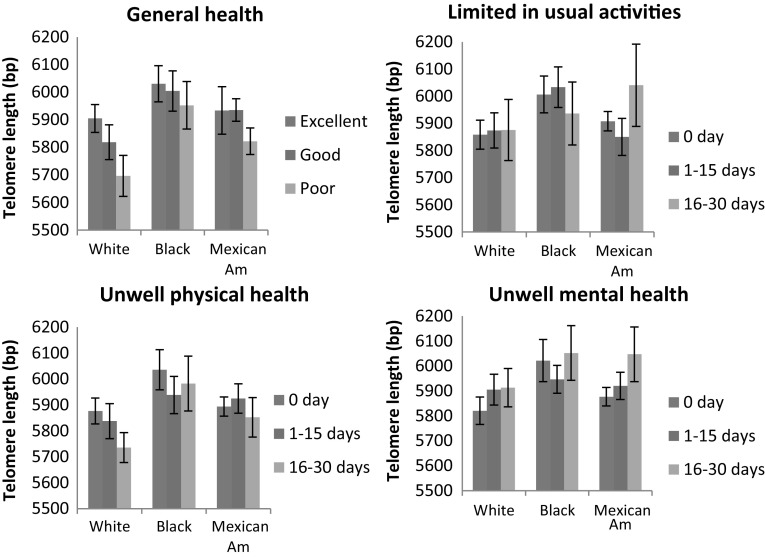
Estimated mean leukocyte telomere length of US adults by race and four dimensions of health-related quality of life (National Health Interview Survey, 2001–2002, *N* = 3547). Base pairs (bp) were calculated using the formula “3274 + 2413 × T/S ratio (telomere length relative to standard reference DNA).” Mean calculated with adjustment for sample weights and design effects. Limit lines indicate 95% confidence interval. Abbreviations, *Mexican Am* Mexican Americans

Figure 1 portrays descriptive relationships between four different dimensions of HRQOL and estimated mean LTL by race. Participants reporting excellent general health had longer LTL compared to participants reporting good or poor general health. This pattern of decreasing LTL with worsening of perceived general health for all Blacks and Whites was distinct in Fig. 1. For perceived physical health, this pattern remains true only for the Whites, where LTL decreased with increased numbers of physically unwell days. No evident pattern was observed for unwell days related to mental health or activity limitation.

The adjusted associations between different HRQOL measures and log-transformed LTL, estimated from multivariate regression models, are presented in Table 3. In age- and demographic-adjusted model, worse perception of general health was significantly associated with shorter LTL for Blacks (regression coefficient, β: −0.021, 95% CI: −0.032 to −0.010, *p* value 0.001), but not for White or Mexican Americans. The association remained significant (β: −0.022, 95% CI: −0.033 to −0.011, *p* value 0.001) after adjustment for other disease and lifestyle variables. Thus, according to the fully adjusted model, having a “good” perception of general health was significantly associated with 2.2% shorter LTL compared to an “excellent” perception about general health for Blacks. No such association was observed for “poor” perception of general health. For Whites, recent days of unwell physical health was associated with shorter LTL. Those having 1–15 physically unwell days in the last 30 days were more likely to have 0.5% shorter LTL than those reporting 0 physically unwell days (β: −0.005, 95% CI: −0.01 to −0.001, *p* value 0.034). Having 1–15 physically unwell days was also marginally associated with shorter LTL for Blacks (β: −0.009, 95% CI: −0.019 to 0.001, *p* value 0.061). However, those reporting 16–30 physically unwell days in last 30 days showed no association with shorter LTL. No associations were observed with other HRQOL measures, such as mental unwell days or days with usual activity limitations for any of the races.

**Table 3:** Association between four dimensions of health-related quality of life measures and log-transformed telomere length in US adults, estimated from multivariate regression models (National Health Interview Survey, 2001–2002, *N* = 3547)

	General health		Unwell physical health during the past 30 days		Unwell mental health during the past 30 days		Activity limitation during the past 30 days	
	β (SE)		β (SE)		β (SE)		β (SE)	
Reference group	Excellent		0 day		0 day		0 day	
	Good	Poor	1–15 days	16–30 days	1–15 days	16–30 days	1–15 days	16–30 days
**Model** **1** ^**a**^								
White	−.007 (.004)	−.001 (.006)	−.006*** (.002)	−.005* (.002)	.0001 (.002)	.002 (.005)	−.003 (.003)	.003 (.007)
Black	−.021*** (.005)	−.007 (.004)	−.009* (.004)	−.002 (.006)	−.006 (.004)	−.005 (.005)	.001 (.002)	−.004 (.005)
Mexican American	−.007 (.004)	−.001 (.009)	.001 (.002)	.010 (.006)	.0001 (.003)	.011 (.006)	−.005 (.003)	.017 (.011)
**Model ** **2** ^**b**^								
White	−.007 (.004)	−.001 (.005)	−.006*** (.002)	-.005* (.002)	.0002 (.002)	.002 (.005)	−.003 (.003)	.004 (.007)
Black	−.021*** (.004)	−.007 (.005)	−.009* (.005)	−.004 (.006)	−.006 (.004)	−.005 (.005)	.001 (.002)	−.004 (.006)
Mexican American	−.007 (.004)	−.0006 (.009)	.002 (.002)	.011 (.006)	−.0001 (.003)	.009 (.006)	−.004 (.002)	.017 (.010)
**Model** **3** ^**c**^								
White	−.007 (.004)	.001 (.006)	−.005** (.002)	−.003 (.003)	.0003 (.002)	.003 (.006)	−.002 (.003)	.007 (.006)
Black	−.022*** (.005)	−.006 (.005)	−.009* (.004)	−.004 (.006)	−.007 (.004)	−.006 (.005)	.0002 (.003)	−.005 (.006)
Mexican American	.002 (.002)	.011 (.006)	−.006 (.004)	.002 (.009)	−.0004 (.003)	.013 (.005)	−.004 (.003)	.018 (.010)

Values are multivariable adjusted β coefficients, with linearized standard errors (SE) in parentheses

Estimated with adjustment for sample weights for the genetic subsample and design effects

Boldface indicates statistical significance (*P* values: *** *p* value ≤ 0.01, ** *p* value ≤ 0.05, * *p* value ≤ 0.10)

^a^Adjusted for age, gender, education, marital status

^b^Adjusted for age, gender, education, marital status, hypertension, diabetes, obesity, cancer status, congestive heart failure

^c^Adjusted for age, gender, education, marital status, hypertension, diabetes, obesity, cancer status, congestive heart failure, smoking status, physical activity, and alcohol intake

## Discussion

The main aim of this study was to determine the nature of the race-specific association between perceived health status as defined by four dimensions of HRQOL-4 and LTL using a large and nationally representative sample while adjusting for an array of potential confounders. Consistent with the hypothesis, our results indicated that negative perception of self-rated general health and unwell days due to recent poor physical health were associated with greater cellular aging as indexed by shorter LTL. One to 15 days of unwell physical health was significantly associated with shorter LTL in Whites, while for Blacks the association was relatively weaker and did not reach a conventional level of significance. The other dimension, “good” perception of general health, was strongly associated with shorter LTL for Blacks compared to “excellent” perception. We, however, found no such relationship for “poor” perception of general health. No association was observed between unwell days due to poor mental health or activity limitations due to poor physical or mental health and LTL for any race.

HRQOL reflects subjective perception of health-related quality, and several community-based studies have reported a relationship between HRQOL and mortality, defined by chronological age at death [5–11]. Most of these studies had participants from a selected group of sample, mainly elderly population or hospitalized and critically ill patients. We not only have extended these findings in a representative general population, but also have used LTL as outcome, which has emerged as a potential biomarker and causative agent of aging at the cellular level [64]. Two dimensions of HRQOL-4, negative perception of general health for Blacks and unwell days due to recent poor physical health for Whites, were associated with shorter LTL in our study. The associations remained after adjusting for several important lifestyle variables and disease conditions. This suggests that the links are independent and perhaps reflect the impact of important economic and psychosocial elements on aging that HRQOL captures beyond objective health assessments. Such elements like interpersonal chronic stressors, varying reaction to health conditions, socioeconomic position, social engagement, economic burden, poor neighborhood conditions, and lack of resources, individually or collectively, can cause repeated and prolonged neuroendocrine, immune, and metabolic regulatory system disruptions causing oxidative stress, inflammation, and inhibition of DNA repair. Since telomeres are particularly sensitive to damage by oxidative stress because of the high guanine content in telomere sequences, this may accelerate leukocyte telomere shortening by promoting cell turnover and replicative senescence [65].

Though perceived general and physical health had the expected relationship with LTL, the relationship with mental health was less consistent and not significant. Perceived stress has been linked to oxidative DNA damage in leukocytes and telomere shortening in [66–69]. Population-based studies have also reported significant association of individual-level psychological factors, such as social stressors, greater anxiety symptoms, and depression with shorter LTL [70]. In contrast to this, we observed no associations between unwell days due to poor mental and LTL. No studies of which we are aware have exactly investigated the association between mentally unwell days and LTL. The closest we found were studies which used the same mental health measure as ours and examined its association with mortality. Interestingly, similar to our findings, they also reported no significant impact of perceived mental health as measured by unwell days count on mortality [71–73]. Taken together with these findings, our results might indicate that as a HRQOL measure, unwell days count past one month probably does not capture the actual stress level or mental health of an individual. The dilution of the strength of association could also be due to recall bias associated with the mental health question. Evidence suggests that one-time cross-sectional population surveys are potentially susceptible to recall bias and may consistently underestimate the prevalence of mental disorders [74]. Alternatively, the underestimation could also be due to the influence of a substantial level of stigma that is still linked to mental disorders [75].

We observed poorer perception of general health to be strongly associated with shorter LTL in Blacks. No association, however, was found in Whites or in Mexican Americans. Having a “good” perception of general health was significantly associated with 2.2% shorter LTL compared to an “excellent” perception about general health, which is roughly equivalent to about 3–5 years of aging on average (calculation provided in the supplementary section) [76, 77]. Since subjective evaluation of general health in HRQOL encompasses a range of economic and psychosocial factors beyond mere physical health conditions, our finding seems to indicate toward a racial disparity in association between these factors and LTL. Few studies have investigated the race differences in telomere length. It is well established that US Blacks have worse health outcomes for most major conditions and have lower life expectancy compared to Whites, but with regard to telomere, a majority of studies reported the opposite direction of this hypothesis, which is a pattern of Blacks having longer LTL than Whites and Mexican American [31, 32, 47–50, 78]. Interestingly, studies have also reported a steeper decline in telomere length with age in Blacks than in Whites [50, 78]. Although LTL is influenced by genetic and epigenetic factors, the differences in the association of age with telomere length by race suggest that environmental factors, which may include factors that increase inflammation or factors that increase oxidative stress, are likely to play an important role in race differences. Economic and psychosocial factors have been linked to inflammation and oxidative stress [41, 79]. Since perception of general health may capture economic and psychosocial factors, our results indicate that a greater exposure to a range of economic and psychosocial factors in Blacks could play a role for the steeper decline in LTL with age in this population.

It is important to note that, while we found an effect for “good” general health (versus “excellent”) on LTL, those who reported “poor” general health did not have shorter LTL than those reporting “excellent” general health. Several factors must be considered when interpreting this inconsistent association between perceived general health status and LTL. For instance, the reason behind this could be the relatively smaller proportion of sample in the “poor” health category. Since the proportion was low, the comparison in the regression analysis probably was not efficient. Alternatively, this finding could be suggestive of complexities of telomere dynamics. It is possible that those with poor health adopted a healthier lifestyle and went through different coping mechanisms, and there are several indications that a healthy lifestyle and stress-coping strategies might alter the rate of telomere erosion [80]. Another important factor that should be considered is, those with poor health could have experienced telomere degradation to such a degree that telomere lengthening pathways could be triggered due to increased levels of telomerase in those undergoing stress due to “poor” perception of health [81]. This inconsistency could also be due to a relatively higher level of albumin and uric acid, two endogenous antioxidants, which could counteract the damaging effect of oxidative stress and attenuate the rapid erosion of telomeres [82]. The relationship between perceived health status and LTL thus remains complex, and future studies should explore other potential mediators, such as diet, history of infection, and exposure to environmental toxins, as well as exposure to stressful environments.

Our findings have several implications and provide a foundation for future exploratory research. The association of HRQOL with mortality and disease morbidity is well documented, yet we know relatively little about the biological mechanisms underlying the association. The relationship between HRQOL and LTL in the current study persisted after adjustment for demographic, disease, and lifestyle variables. Therefore, our results also indicate that telomeric attrition attributable to health-related psychosocial elements that HRQOL represents could be an important mechanism underlying the association between perceived health and morbidity or mortality. The inconsistency in association between unwell days due to poor mental health and LTL deserves reassessing this question as an indicator of mental health. Analyses of unwell day measures should be taken with caution however, given the possibility of recall bias (asked about past 30 days). Thus, there is a possibility of non-differential misclassification which might have resulted in an underestimate of the true strength of the association. Further research will aid in understanding the relative contribution, potential modification, and efficient use of HRQOL measure in assessing precise future health risks. Our data provide evidence of a possible stronger cross-sectional association of perceived general health and LTL shortening in Blacks than in Whites and Mexican Americans. The precise mechanism and determinants of this disparity remain to be identified but could involve greater exposure to a range of economic and psychosocial factors over the lifecourse in Blacks compared to other races. We recommend additional multiethnic studies to confirm this and to understand the reasons and consequences of this disparity.

## Limitations

Our results should be interpreted within the context of a few limitations. We acknowledge that given our cross-sectional observational design, our study can only examine the associations of the HRQOL with LTL, but precludes drawing causal inferences. That is, rather being a direct or indirect causal factor in telomere shortening, poor HRQOL may occur as a result of the pathophysiological effects of telomere shortening itself. It is also important to note that with only one time-point telomere data, we could not account for innate individual variation in telomere length. Therefore, longitudinal studies with repeated measures of LTL are needed to determine whether the observed associations are causal and, if so, to identify the specific mechanisms involved. Our findings may also be limited by residual confounding due to limited accuracy in the measures of available variables. For example, absence of measures of lifestyle variables over the lifecourse may have limited our ability to adjust for these factors. LTL is strongly influenced by genetic factors. Hence, it is possible that genetic factors could also modulate the associations of HRQOL with LTL. Our findings may also be limited by other unmeasured variables, such as diet, psychological stress, neighborhood conditions, and proportion of different leukocyte subtypes. All of these variables could contribute to population differences in LTL. Additional studies should consider these factors, and the role of genetic variants, and elucidate the underlying mechanisms of the associations between HRQOL and LTL.

## Conclusion

In conclusion, though we did not find a gradient relationship in the multiple regression models, negative perception of self-rated general health and unwell days due to recent poor physical health were associated with greater cellular aging as indexed by shorter LTL above and beyond individual risk factors. Thus, our findings contribute evidence to the limited literature that HRQOL, as a source of economic and psychosocial burden, could be associated with LTL shortening. Although longitudinal studies are needed to better understand and confirm this relationship, our results, which come from a large nationally representative sample across a wide range of age, provide preliminary evidence that HRQOL could be associated with LTL shortening. We also found a possible racial difference in this association and recommend additional multiethnic studies to confirm this and to understand the reasons and consequences of this difference.

## Electronic supplementary material

Below is the link to the electronic supplementary material.

Supplementary material 1 (DOCX 62 kb)
