# Supplementary material for: Race-specific associations between health-related quality of life and cellular aging among adults in the United States: evidence from the National Health and Nutrition Examination Survey
Source: Qual Life Res. 2017 Jun 8;26(10):2659–69. doi: 10.1007/s11136-017-1610-9 (PMC5597687; doi:10.1007/s11136-017-1610-9)
Supplement: Supplementary file 1 — Supplementary material 1 (DOCX 62 kb) [file 11136_2017_1610_MOESM1_ESM.docx]

**Article title:** Race-specific associations between health-related quality of life and cellular aging among adults in the United States: Evidence from the National Health and Nutrition Examination Survey

**Journal name:** Quality of Life Research

**Author names**

Rumana J Khan

Samson Y Gebreab

Pia R Crespo

Ruihua Xu

Amadou Gaye

Sharon K Davis

**Affiliation of the authors**

Authors are from the Genomics of Metabolic, Cardiovascular and Inflammatory Disease Branch, Social Epidemiology Research Unit, National Human Genome Research Institute, National Institutes of Health, Bethesda, Maryland, USA

**Corresponding author**

Rumana J Khan

e-mail address: rumana.khan@nih.gov

**Analytical methods for telomere**

Aliquots of purified DNA were provided by the laboratory at National Center for Health Statistics. DNA was isolated from whole blood using the Puregene (D-50K) kit protocol (Gentra Systems, Inc., Minneapolis, MN) and stored at −80 °C. The leukocyte telomere length (LTL) assay was performed in the laboratory of Dr. Elizabeth Blackburn at the University of California, San Francisco, using the quantitative polymerase chain reaction method to measure LTL relative to standard reference DNA (also known as the T/S ratio). [[1](#_ENREF_1)] For quality control, each sample was assayed 3 times on 3 different days. The samples were assayed on duplicate wells, resulting in 6 data points. Sample plates were assayed in groups of three plates, and no two plates were grouped together more than once. Each assay plate contained 96 control wells with 8 control DNA samples. Assay runs with 8 or more invalid control wells were excluded from further analysis (< 1% of runs). Control DNA values were used to normalize between-run variability. Runs with more than 4 control DNA values falling outside 2.5 standard deviations from the mean for all assay runs were excluded from further analysis (< 6% of runs). For each sample, any potential outliers were identified and excluded from the calculations (< 2% of samples). The mean and standard deviation of the T/S ratio were then calculated normally. The interassay coefficient of variation was 6.5%. [[2](#_ENREF_2)] DNA samples were coded and the lab was blinded to all other measurements in the study. The CDC Institutional Review Board provided human subject approval for this study. The conversion from T/S ratio to base pairs (bp) was calculated based on comparison of telomeric restriction fragment length from Southern blot analysis and T/S ratios using DNA samples from the human diploid fibroblast cell line IMR90 at different population doublings. The formula to convert T/S ratio to bp was 3274+2413 × (T/S). [[3](#_ENREF_3), [1](#_ENREF_1), [2](#_ENREF_2)]

**References**

1. Lin, J., Epel, E., Cheon, J., Kroenke, C., Sinclair, E., Bigos, M., et al. (2010). Analyses and comparisons of telomerase activity and telomere length in human T and B cells: Insights for epidemiology of telomere maintenance. *Journal of Immunological Methods, 352*(1-2), 71–80.

2. 2001 - 2002 Data Documentation, Codebook, and Frequencies, Telomere Mean and Standard Deviation (Surplus) (TELO_B), National Health and Nutrition Examination Survey (2014). National Health and Nutrition Examination Survey.

3. Cawthon, R. M. (2002). Telomere measurement by quantitative PCR. *Nucleic Acids Research, 30*(10), e47-e47.

**Supplementary chart 1.**

Flow chart of study participants (National Health Interview Survey, 2001–2002)

5411 eligible participants

Did not provided DNA/did not consent to its use in future research/did not have a sufficient quantity of DNA to estimate telomere length

Excluded 1151

4260 participants with telomere data

No data on health related quality of life to participate

Excluded 270

3990 participants had information on HRQOL

Missing data on educational level (4), marital status (1), disease status or presence of hypertension, diabetes, cancer, orheart failure (36), body mass index (154), and alcohol consumption (1)

Excluded 196

3794 participants had information on all variables

Race was not specified and were not considered for the analysis

Excluded 247

Final sample for analysis = 3547 participants

**Note:** The National Health and Nutrition Examination Survey (NHANES), a representative sample of the United States population, randomly selects 5000 individuals each year to participate in the survey. The overall response rates are usually very high and similar for all races. However, regarding genetic research (telomere in our study) Blacks, women, and subjects older than 60 years of age were less likely to give consent for future genetic research. In our study sample about 21% (1151 out of 5411) did not have telomere data.

These are considered as non-response and weights are created in NHANES to account for complex survey design (including oversampling) as well as survey non-response. We used NHANES-specified weights for the bio specimen variables. Since weights account for survey non-response, here missing value treatment was not needed.

**References**

1. McQuillan, G. M., Pan, Q., & Porter, K. S. (2006). Consent for genetic research in a general population: An update on the National Health and Nutrition Examination Survey experience. *Genetics in Medicine*, *8*, 354–360.
2. National Health and Nutrition Examination Survey, 2001 - 2002 Data Documentation, Codebook, and Frequencies, Telomere Mean and Standard Deviation (Surplus) (TELO_B) (2014). National Health and Nutrition Examination Survey.
3. National Health and Nutrition Examination Survey Analytic and Reporting Guidelines. (2004). <http://www.cdc.gov/nchs/data/nhanes/nhanes_general_guidelines_june_04.pdf>

**Supplementary chart 2.**

Four measures of Health-Related Quality of Life (HRQOL) questionnaire and their categorization for analysis

**Now thinking about your *physical health*, which includes physical illness and injury, for how many days during the past 30 days was your physical health not good?**

Total number of unwell was grouped into 0, 1 to 15, and 16 to 30 days, where 0 day served the reference group

Missing data for hip circumference (5)

**Would you say that your *general health* is: excellent, very good, good, fair, or poor?**

Re-categorized for analysis as:

Excellent (excellent + very good),

Good (good)

Poor (fair+ poor), with “excellent” being the reference

Health-Related Quality of Life

**During the past 30 days, for about how many days did poor physical or mental health keep you from doing your *usual activities*, such as self-care, work, or recreation?**

Total number of unwell was grouped into 0, 1 to 15, and 16 to 30 days, where 0 day served the reference group

**Now thinking about your *mental health*, which includes stress, depression, and problems with emotions, for how many days during the past 30 days was your mental health not good?**

Total number of unwell was grouped into 0, 1 to 15, and 16 to 30 days, where 0 day served the reference group

**Health related quality of life-race interaction findings**

**Supplementary Table** 1 β coefficients, 95% conf. intervals, and p values of interaction terms (multiplicative) for physical health and general health with race estimated from multivariate regression models (National Health Interview Survey, 2001–2002, N=3547)

|  | β | 95% Conf. Interval | P value |
| --- | --- | --- | --- |
| Race*physical health 1 | 0.003 | 0.0005 to 0.007 | 0.03 |
| Race*physical health 2 | 0.008 | -0.002 to 0.0179 | 0.10 |
| Race*general health 1 | -0.0066 | -0.013 to -0.0006 | 0.032 |
| Race*general health 2 | -0.0036 | -0.011 to 0.0034 | 0.20 |

physical health 11 to 15 unwell days; and physical health 2= 16 to 30 unwell days (0 unwell day is the reference)

general health 1= good general health; and general health 2= poor general health (excellent general heath is the reference)

Adjusted for age, gender, education, marital status, hypertension, diabetes, obesity, cancer status, congestive heart failure, smoking status, physical activity, and alcohol intake

**F tests results comparing full and reduced models (with and without the interaction term)**

Ftest Model_1 Model_2

Assumption: Model_1 nested in Model_2

F (8, 3650) = 1.819

prob > F = 0.066

Model_1 = no interaction terms

Model_2 = with interaction terms (health related quality of life measures * race)

**Leukocyte telomere length and age in adults**

A yearly telomere loss of 24.7 to 45.7 base pairs (BP)/year was estimated by weighted regression

Assuming an adult having ~6000 BP corresponds to 132 BP of loss at a rate of 2.2% (2.2*6000/100)

If we assume a yearly telomere loss of 24.7 to 45.7 BP/year, 132 BP is equivalent to 3 to 5 years of life

**References**

1. Marioni, R. E., Harris, S. E., Shah, S., McRae, A. F., von Zglinicki, T., Martin-Ruiz, C., et al. (2016). The epigenetic clock and telomere length are independently associated with chronological age and mortality. *International Journal of Epidemiology*, *45* (2), 424–432.
2. Muezzinler, A., Zaineddin, A. K., & Brenner, H. (2013). A systematic review of leukocyte telomere length and age in adults. *Ageing Research Reviews*, *12*(2), 509–519.
